# Ayurveda in the integrative management of bovine secondary recumbency-a case report

**DOI:** 10.1016/j.jaim.2024.100962

**Published:** 2024-08-08

**Authors:** Reshma R, Unnikrishnan K

**Affiliations:** a P. G Scholar, Department of Shalakya, Amrita School of Ayurveda, Kollam, Kerala, India; b Veterinary Officer (Retd), Kerala. Managing Director, Apollo Multi-specialty Animal Hospital, Changanacherry, Kerala, India

**Keywords:** Downer cow, Pashu ayurveda, Ayurvedic veterinary, Ruminal atony, Non-ambulatory

## Abstract

Downer cow syndrome, or secondary recumbency, is a condition primarily affecting dairy cows, where the animal is unable to rise and stand, due to unknown cause. It is usually associated with poor prognosis. Terminal downers are euthanized in most countries. A four-year old *Kasarkode* dwarf-cow, post-calving was brought up laterally recumbent with heavy nasal discharge, labored breathing, loss of appetite and signs of dehydration on 6th day of its recumbency. Before this, the patient was diagnosed with milk fever and standard treatment with calcium borogluconate was administered intravenously. The animal was left to succumb under unprotected conditions, due to various constraints on euthanasia. After adopting the cow, *Nasya* was started immediately to avoid death due to sepsis and shock. The animal was drenched with Ayurvedic fluids containing *deepana-pacana* herbs. Sternal recumbency, warm and moistened muzzle was observed on the fourth day of commencing ayurvedic treatment. Respiratory distress was minimal. Drastic prognostic shift from “no hope” to “good” was possible within 6 days thanks to Nasya, and the animal was stable. Thereafter, integrative care comprising of antibiotics, rehydrating IV fluids, and supplementations, along with ayurvedic medicines was initiated. Ruminal-fluid obtained from slaughterhouse was used for ruminal-flora replacement. Rumination on 14th day, cow on its feet by 19th day and complete healing of decubital ulcers by approximately 40 days was recorded. A downed cow which did not respond to standard veterinary care was managed with Ayurveda-integrated veterinary care. Ayurveda herbs like bamboo leaves (*Bambusa vulgaris*), green chiretta (*Andrographis paniculata)* that cattle prefer eating during certain illness, turn out to be useful for Ayurvedic management. Hence, Ayurveda veterinary medicine might be, a good choice for integrative management of terminal downers, preventing early death in downed dairy cows.

## 1 Introduction

Bovine secondary recumbency is conversationally called “downer cows” where the animal fails to stand on its feet, involuntarily. Downer dairy cows are more likely to be separated and culled within 30 days of diagnosis [1]. Several causes like infections, metabolic insults, trauma, and degenerative conditions for recumbency have been documented in cows [2]. When these causes are directly related, it is termed as primary recumbency and if the condition is not treated, or unsuccessfully managed, it can result in secondary recumbency after 24 h. In such prolonged recumbency, the cow will be prone to both muscle and nerve damage due to pressure-related injuries, firstly due to its own body weight on the muscle it is resting, and secondly due to compartment syndrome. This apparently results from venous engorgement and reduced lymphatic drainage, due to continuous pressure on the muscle, nerves, and blood vessels within the fascia owing to extravasation of fluid. Additionally, decubital ulcers and trauma to the muscles, nerves, bones and joints, tendons can make the condition painful for the animal. Sciatic nerve injury can prolong the time of recumbency, for these downers. Therefore, it's a challenge for both the farmer and local veterinarian since the outcome of treatment is quite at times unpredictable.

The branch of Ayurveda dealing with cows, Pashu ayurveda [3], elaborates various treatment protocols for terminally ill cows. Time and time again, these animals become stray cattle without food and water because of poor response to standard treatment. This case report presents a simple method of managing downer cows, integrating ayurveda with standard veterinary management.

## 2 Case report

### 2.1 Patient information

A four-year old *Kasarkode* dwarf-cow, post-calving, (second parity) was brought up laterally recumbent with heavy nasal discharge, tachypnea, dyspnea, loss of appetite and signs of dehydration on 6th day of recumbency. The animal did not have dystocia, and patient expelled placenta few hours after calving, was found standing post calving, and the calf also drank its colostrum as reported by the cattleman. The animal refused to forage and became an alert-downer, 10 h post calving. Standard veterinary treatment for milk fever was attempted. This included treatment for hypocalcemia and hypophosphatemia, and supportive care, based on lab reports on serum electrolytes, (reported by the local veterinarian). Within 5 days, the condition progressed to acute respiratory distress. It was left unprotected under rain since emergency euthanasia isn't a common practice.

### 2.2 Clinical findings

The animal was brought lying laterally recumbent, weak, with no signs of external trauma. Animal appeared dull and depressed, hypothermic (88°F- rectal) with a cold and dry muzzle, labored breathing ∼ 56/min with sound, slow heart rate, no rumination, unresponsive to external environment, with sunken eyes. No signs of birth trauma (swelling or infection) or vaginal discharge, prolapse of tissues from vulva was noted. No signs of mastitis were observed. The animal was completely dehydrated. Ruminal movements almost nil (left flank) during palpation. The cow drenched very little water, infrequently. On day 6, the veterinarian diagnosed the case as Downer cow syndrome with total ruminal atony, rumen acidosis - pH 5.0.(ruminal pH after paracentesis) and fever, (103°F- rectal).

**Timeline** - Details as given in Table no 1.

### 2.3 Diagnosis

Usually, clinical findings with bedside examination are sufficient to arrive at a diagnosis. However, it is important here to note patient treatment history. Prior to admission, the local veterinarian had sent samples for blood routine, and lab report showed low calcium and low phosphorous levels. The animal was diagnosed with milk fever, and despite standard treatment with calcium borogluconate therapy, the animal showed no response. On admission, ayurveda diagnosis was derived from Sahadeva’ text on Pashu ayurveda. From the text, the cattle refusing to eat, and drink was diagnosed as Neridi, Domma and/or Nallatevulu [3] and the veterinarian diagnosis was ruminal atony, on the 6th day of Ayurvedic treatment.

## 3 Therapeutic intervention

### 3.1 Ayurvedic treatment

Nasya – intranasal instillation drops made from sesame oil, *grhadhuma* (chimney soot) and Italian millet (*Setaria italica*) 30 drops in each nostril was given three times daily for three days. The animal was moved to a soft wood chip bedding. The animal was drenched with juice of bamboo leaves (*Bambusa vulgaris*) 30ml, twice daily. The animal was also drenched with solutions ∼400 ml, four times daily, with appetite inducing powder of drugs like Triphala [4] *(*fruits of *Emblica officinalis, Terminalia bellerica, and Terminalia chebula)* Trikatu [5] (*Piper longum Linn, Zingiber officinale, Piper nigrum*) with juice of tamarind (*Tamarindus indica*); leaf pulp of aloe vera (200 ml) was added with jaggery (100 g) and was administered for 12 days. This administration of aloe vera with jaggery is a common practice among cattlemen, to reduce patient's inflammatory ailments and to meet energy requirements. Belly bands were used to lift the cow to keep it standing on feet for 20 min but was stopped when the skin started to bruise near the bands (Fig. 3). The shed was fumigated everyday with *Guggulu* (*Commiphora wightii*) for its wound healing and anti-microbial activity [6]. The cow was supported with pillows to keep it propped up and prevent pressure damage to muscles and nerves. The cow was also repositioned every 3 h for this purpose. Externally, *Sahacharadhi thailam,* (75 ml) was applied and hot fomentation was done on all four limbs, to alleviate musculoskeletal pain when the cow started shifting its positions [7].

### 3.2 Standard veterinary care

From day 6, treatment was integrated with standard veterinary care. Anti-biotics, IV fluids for fluid and electrolyte imbalance, steroids, were started, as per standard protocol. Fluid replacement was done with normal saline 1 L, for 3 days with sodium bicarbonate 5% solution 200 ml for 3 days. Prednisolone 10ml/I. M and methyl prednisolone depot 3 ml epidural was administered. Administration of Thiamine (Beplex forte) I. M, 8 ml/24 hourly for 3 days and Inj. Enrofloxacin L.A (Fortivir) 10 ml was given I. M for 24 hourly for 3 days. Butaphosphan and Cyanocobalamin intravenous Injection, 10 ml for 3 days, Inj. Calcium borogluconate (Borocal) 200 ml for 2 days was given intravenously. Decubitus ulcers were treated with combination of Zinc oxide, iodoform and paraffin paste. The cow was fed with ruminal fluid ∼1500 ml obtained from slaughterhouse and the ruminal fluid was fed immediately after its collection, for three days as ruminal transfaunation.

### 3.3 Outcome

**Fig. 1:**
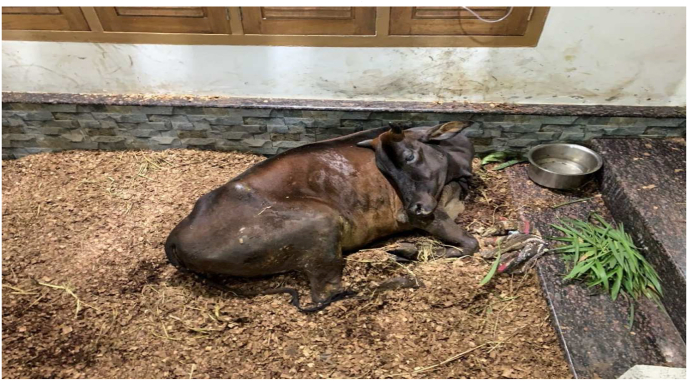
Downer cow, on Day 4 of treatment.

**Fig. 2:**
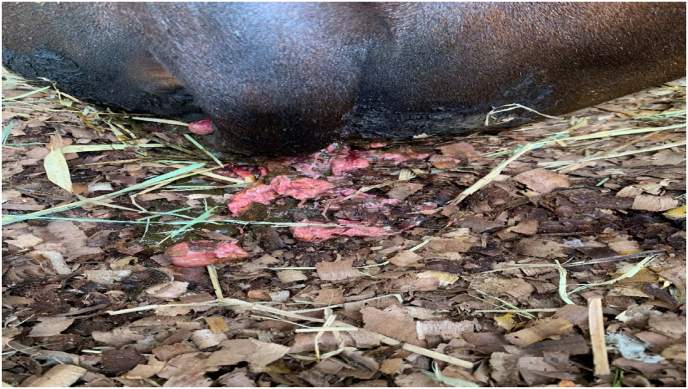
Copious vaginal discharge.

The animal was laterally recumbent on the first day when *Nasya* was administered. The patient head was kept elevated during and after the procedure to prevent backflow of the drops instilled. Heavy mucoid nasal discharge was seen on day 2 and day 3. On day 4, the animal returned to sternal recumbency, with head turned towards to its side (Fig. 1). It started to smell the leaves. On day 5, the nasal discharge was minimal. Breathing distress was also moderately controlled. Animal had copious urine output and smelly vaginal discharge (Fig. 2). On day 6, the hydration status remained good after administration of IV fluids. On day 10, the cow started eating 1–2 bamboo leaves and green chiretta leaves, breathlessness was mild and rumination for few hours started on day 13. Rumination increased over time and the cow began licking its muzzle and nostril on day 14. The cow crawled on its fore limb and started to change its positions on day 16. The cow started eating and drinking enough. The cow stood on its feet on day 19. Staggering gait was observed by Day 22 (Fig. 4). Treatment was completed by Day 22. Decubitus wound healing was noted by day 25 (Fig 5) and the animal looked healthy on feet by day 40. However,there was no alteration in the lactation status; the animal remained non-lactating. Table 1 shows the treatment timeline of the patient.

**Fig. 3:**
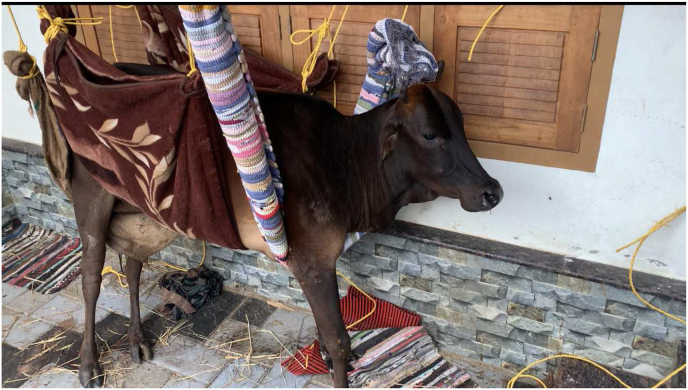
Attempt to support cow with belly bands for reducing pressure induced muscle damage.

**Fig. 4:**
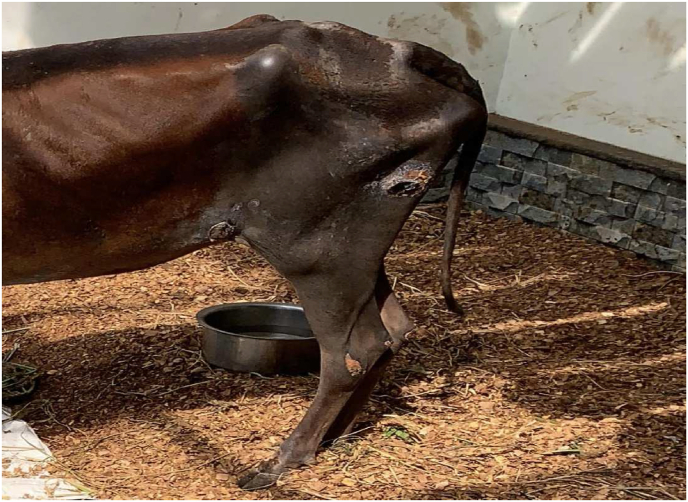
Decubitus ulcers.

**Fig. 5:**
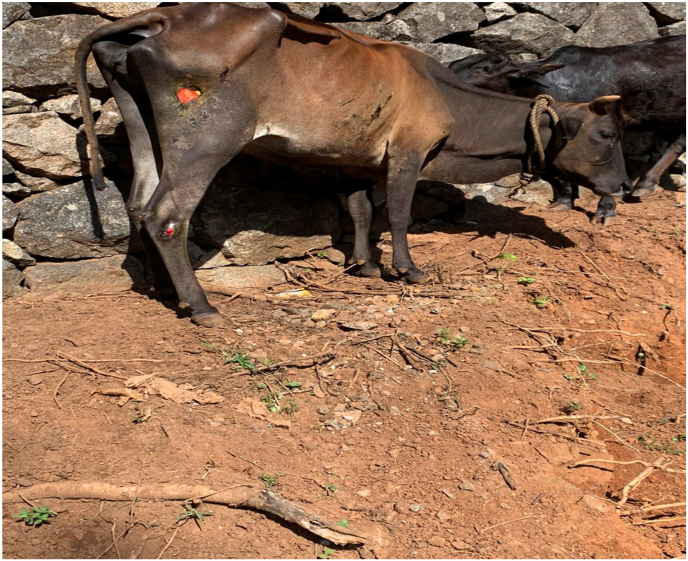
Cow walking on ground - 23rd day.

**Table 1:** Treatment timeline of the patient.

Day of Treatment	Patient Symptoms	Pashu ayurveda chikitsa	Veterinary treatment
1st - 3rd	Lateral recumbency Heavy mucoid nasal discharge, dull and depressed, hypothermic (88°F- rectal) with a cold and dry muzzle, laboured breathing ∼ 56/min with sound, slow heart rate, no rumination, unresponsive to external environment, with sunken eyes.	Nasya with drops made from sesame oil, *grhadhuma* (chimney soot) and Italian millet (*Setaria italica*) 30 drops in each nostril was given three times daily for first 3 days. Drenched with 1. Juice of bamboo leaves (*Bambusa vulgaris*) 2.Appetite inducing powder of drugs like Triphala (fruits of Emblica officinalis, Terminalia bellerica, and Terminalia chebula) Trikatu (Piper longum Linn, Zingiber officinale, Piper nigrum) with juice of tamarind (Tamarindus indica) 400 ml, four times daily 3.Leaf pulp of aloe vera (200 ml) with jaggery (100g) for 12 days.	–
4th and 5th	returned to sternal recumbency nasal discharge reduced		
6th	Symptoms of Downer cow syndrome with total ruminal atony, rumen acidosis - pH 5.0.(ruminal pH after paracentesis) and fever, (103°F- rectal)		Fluid replacement: normal saline 1 L for 3 days with soda bicarbonate 5% solution 200 ml for 3 days. Prednisolone 10ml/I.M and methyl prednisolone depot 3 ml epidural Thiamine (Beplex forte) I.M, 8 ml/24 hourly for 3 days and Inj. Enrofloxacin L.A (Fortivir) 10 ml was given I.M for 24 hourly for 3 days. Butaphosphan and Cyanocobalamin intravenous Injection, 10 ml for 3 days, Inj. Calcium borogluconate (Borocal) 200 ml for 2 days was given intravenously.
10th −19th	Rumination started	The shed was fumigated everyday with *Guggulu* (*Commiphora wightii*) for its wound healing and anti-microbial activity. Externally, *Sahacharadhi thailam*, (75 ml) was applied and hot fomentation was done on all four limbs, to alleviate musculoskeletal pain.	External application of Zinc oxide, iodoform and paraffin paste for decubitus ulcers. Ruminal fluid ∼1500 ml for three days as ruminal transfaunation.
16th	Creeper cow		
19th	Standing on feet		
20th – 22nd	Staggering gait		
23rd- 40th	Normal gait, healing decubitus ulcers	–	–

## 4 Discussion

### 4.1 Current scenario

1-2 cows per 100 cows have the risk of becoming downers [8]. with survival rate recorded at 37%. [9]Usually downer cows, which is an alert, on sternal recumbency, responding to calcium therapy post calving, without loss of appetite have good chances to complete recovery. Treatment or euthanasia depends on many prognostic factors like muscle enzymes, duration of recumbency, muscle damage, age, parity, and quality of care given. Treatment involves two courses of calcium borogluconate therapy, symptomatic treatment, and supportive care with physiotherapy. The prognosis of secondary recumbency is poor. It is associated with much economic loss for the farmer as the animal has neither lactation nor can enter the food chain. Consequently, the animal is mercilessly abandoned. Usually, euthanasia is performed in many countries to prevent animal suffering, with captive bolt pistol, light caliber pistol or rifle or by barbiturate overdose. The major animal welfare problem, however, lies in brutal dragging of diseased cattle with inappropriate equipment, for transportation, topping the list.

### 4.2 Ethno-veterinary medicine in India

Animals especially, cows are considered as an important economic factor in India since time immemorial. Ethno-botanical/veterinary medicine is explained on *Vrkshayurveda* (deals with plant medicine) *Hastyayurveda/Gajayurveda* (medicine for elephants), *Asva/Hayayurveda* (medicine for horses) and *Gavayurvcda* (deals with medicine for cows) in detail in *Garudapurana*. Schwabe in 1978 has described that the first veterinary hospital existed during Ashoka's regime and the *‘Baniyan Hospital’* of *Suratis* is another example of its kind [10].

### 4.3 Ayurveda treatment and rationale

According to *Sahadeva Pashu-ayurveda*, when the cow stops food, water, and rumination, it suffers from *Neridi*. *Nasya* is the treatment of choice. Labored breathing was considered under the disease, *Domma*. In this condition, the animal is orally administered *Triphala, Trikatu* with tamarind juice. In a disease called *Nallatevulu*, the cattle are given juice of *Andrographis paniculata* and bamboo leaves. *Nallatevulu* is synonymous with *Domma.* Since a direct reference was unavailable for downer cow, the treatments mentioned under these topics, were administered.

Traditionally, *Nasya* is indicated in diseases that have localized above the shoulders. Also, in addressing emergency situations like bronchial asthma [11]. Here the usage of Nasya in the treatment of complete loss of appetite, is worth exploring. Perhaps,the concept of post-partum *shvayathu* can be contemplated for disease *samprapti*. Downer cow syndrome is considered a metabolic problem, generally and *Triphala* is an important drug of choice in metabolic insults. *Triphala* has ellagic acids, gallic acid, tannins, chebulagic acid, phenols, and glycosides [12]. It stimulates appetite, a potent antioxidant, anti-inflammatory, antibacterial, antimutagenic, hypoglycemic, antineoplastic, with immunomodulating effects [13]. *Trikatu* is a bio-availability enhancer, and helpful in improving the concentration of the accompanying drugs. Levels of glutamic oxalacetic transaminase, serum pyruvic transaminase and alkaline phosphatase were substantially higher in downer cows [14]. Intake of tamarind pulp was known to reduce the level of Zn dependent enzymes like alkaline phosphatase [15] Aloe vera was found to have *anti*-hyperlipidemic and anti-inflammatory antioxidant effects especially helping liver and kidney health on transition dairy cows in early lactation period [16].

## 5 Conclusion

The need to realize the multiple scopes of this case report is not only necessary but crucial to having a “*May the whole world be happy”* mantra in our minds. To preserve our native cows, to implement “*Pashu Ayurveda*” in treating cattle diseases, to reduce the economic burden of

cattleman and ultimately to reduce the number of animals slaughtered in its youth represents some of the achievable goals projected in this paper. This is simply possible with management of downer cows, integrating Ayurveda.

## Declaration of generative AI in scientific writing

No AI tools/services were used during the preparation of this work.

## Source(s) of funding

None.

## Author Contributions

Reshma R was responsible for writing original draft, Unnikrishnan K contributed in review and editing. Both authors read and approved the final draft.

## Declaration of competing interest

The author declares no conflict of interest, financial or otherwise.
